# Exploring Recent Developments in Graphene-Based Cathode Materials for Fuel Cell Applications: A Comprehensive Overview

**DOI:** 10.3390/molecules29122937

**Published:** 2024-06-20

**Authors:** Somya Samantaray, Debabrata Mohanty, Santosh Kumar Satpathy, I-Ming Hung

**Affiliations:** 1 Department of Physics, School of Applied Sciences, Centurion University of Technology and Management, Bhubaneswar 752050, India; 230506162002@centurionuniv.edu.in; 2 Department of Chemical Engineering and Materials Science, Chang Gung University, Taoyuan 333323, Taiwan; debabratamohanty@cgu.edu.tw; 3 Center for Sustainability and Energy Technologies, Chang Gung University, Taoyuan 333323, Taiwan; 4 Department of Chemical Engineering and Materials Science, Yuan Ze University, Taoyuan 32003, Taiwan; 5 Hierarchical Green-Energy Materials (Hi-GEM) Research Center, National Cheng Kung University, Tainan 70101, Taiwan

**Keywords:** fuel cells, SOFC, graphene, triple conductivity, chemical stability

## Abstract

Fuel cells are at the forefront of modern energy research, with graphene-based materials emerging as key enhancers of performance. This overview explores recent advancements in graphene-based cathode materials for fuel cell applications. Graphene’s large surface area and excellent electrical conductivity and mechanical strength make it ideal for use in different solid oxide fuel cells (SOFCs) as well as proton exchange membrane fuel cells (PEMFCs). This review covers various forms of graphene, including graphene oxide (GO), reduced graphene oxide (rGO), and doped graphene, highlighting their unique attributes and catalytic contributions. It also examines the effects of structural modifications, doping, and functional group integrations on the electrochemical properties and durability of graphene-based cathodes. Additionally, we address the thermal stability challenges of graphene derivatives at high SOFC operating temperatures, suggesting potential solutions and future research directions. This analysis underscores the transformative potential of graphene-based materials in advancing fuel cell technology, aiming for more efficient, cost-effective, and durable energy systems.

## 1. Introduction

The demand for clean energy sources is escalating due to the rapid depletion of reserves of fossil fuel along with the pressing need to lower global carbon liberation. Hydrogen is widely regarded as the optimal fuel for the forthcoming decades, with ongoing advancements in hydrogen energy aimed at accelerating its transition from theoretical potential to practical real-world applications. Combining renewable energy resources with cutting-edge fuel carriers and waste-to-energy management strategies [1] offers a promising approach to tackling the energy crisis and the escalating threat of global warming. Liquid hydrogen (LH_2_) arises as a viable substitute to conventional fossil fuels, boasting several advantages such as its lightweight nature, reduced fuel transportation burden [2], high specific energy [3], zero CO_2_ emissions [4], minimal NO_x_ emissions [5], reduced air pollution [6], and environmental sustainability [7], in spite of challenges like high cost and infrastructural disadvantages. Various chemical processes, such as steam methane reforming and methane pyrolysis, can be employed to produce hydrogen. Research works to overcome these limitations are ongoing.

Fuel cells correspond to a class of energy-converting devices capable of generating electricity as long as fuel is available. Compared to combustion engines, fuel cells boast lower pollution emissions and greater system efficiency, as they directly transform the chemical energy of fuels into electricity without the need for an intermediate combustion step. Consequently, fuel cells are recognized as a promising technology to address global energy and environmental concerns, enhancing the sustainability and cleanliness of our lives. Typically, a fuel cell comprises an electrolyte layer sandwiched between two electrodes. At the anode, fuel oxidation occurs, releasing electrons that traverse the externally connected circuit to the cathode, where they reduce oxygen. Concurrently, charge carriers such as H^+^, OH^−^, CO_3_^2−^, or O_2_^−^ pass through the electrolyte. Fuel cell types, including phosphoric acid fuel cells (PAFCs), molten carbonate fuel cells (MCFCs), alkaline fuel cells (AFCs), polymer electrolyte membrane fuel cells (PEMFCs), direct alcohol fuel cells (DAFCs), and solid oxide fuel cells (SOFCs), are categorized based on the types of electrolytes as well as the fuel utilized [8]. Similarly, according to operating temperature, fuel cells are categorized into the following types: (1) alkaline fuel cells, (2) proton exchange membrane fuel cells, (3) direct methanol fuel cells, (4) phosphoric acid fuel cells, (5) molten carbonate fuel cells, and (6) solid oxide fuel cells [9]. Various types of fuel cell categories are distinguished in Table 1.

In general PAFC, PEMFC, and AFC operate at relatively low temperatures (below 200 °C) and are well suited to stationary applications. On the other hand, SOFCs function at temperatures between 600 and 1000 °C and provide advantages such as a lengthy lifespan, minimal noise, reduced carbon dioxide emissions, high conversion rates, and a variety of fuel sources [13]. Low-temperature fuel cells, such as proton exchange membrane fuel cells (PEMFCs), require a hydrogen-rich fuel feed with very low levels of lethal elements like carbon monoxide (less than 20 ppm) and sulfur compounds (at ppm levels); in contrast, high-temperature fuel cells, such as solid oxide fuel cells (SOFCs), are tolerant to carbon monoxide and can utilize methane as a fuel instead of hydrogen. In H-SOFCs, hydrogen is fed into the anode, where it undergoes an oxidation reaction, producing electrons according to the following reaction: 2H_2_ → 4H⁺ + 4e⁻. The electrons generated are released to the external circuit, creating a direct current (DC). Simultaneously, the H⁺ ions produced are transported through the proton-conducting membrane to the cathode. The electrolytes in H-SOFCs must be sufficiently proton-conductive to facilitate this transport [14]. At the cathode, the protons react with oxygen molecules and electrons from the external circuit, forming water vapor. Unlike oxide ion fuel cells, H-SOFCs do not generate water molecules at the anode side, which is an advantage because this eliminates the need for fuel circulation, keeping the anode fuel pure. Hydrogen for the anode can be sourced from various origins, including fossil fuels (petroleum, natural gas, and coal), chemical compounds (methanol and ethanol), syngas from biomass and waste gasification, biofuels from biomass, pyrolysis gas, and renewable energy sources like solar, wind, and geothermal energy, which produce hydrogen via water electrolysis [15]. PEMFCs and DMFCs are considered ideal power sources due to the availability of fuel sources, portability, low operating temperature, and minimal-to-zero emissions of pollutants. Both PEMFCs and DMFCs consist of an anode and a cathode, along with an electrocatalyst that accelerates the electrochemical process. The selection of fuel cell components faces challenges related to electrochemical performance, efficiency, and durability [16]. The composition and structure of the electrodes in fuel cells differ, particularly when comparing PEMFCs, DMFCs, and SOFCs. Both PEMFCs and DMFCs operate at moderate temperatures with a Nafion polymer membrane, and both use platinum-based catalysts on carbon supports for the anode and cathode. PEMFCs use hydrogen as fuel, whereas DMFCs use methanol. In contrast, solid oxide fuel cells (SOFCs) use a solid oxide electrolyte (YSZ) to conduct oxygen ions and can run at high temperatures with a nickel–yttria-stabilized zirconia (Ni-YSZ) anode and a perovskite cathode like lanthanum strontium manganite (LSM). Hydrocarbons and hydrogen can also be used as fuels in SOFCs. Each fuel cell type’s unique operating requirements and fuel types are reflected in these variations [17].

Graphene and its derivatives emerge as attractive materials for fuel cell applications owing to their superior mechanical, electrical, and chemical properties, with significant efforts focused on maximizing their potential applications in fuel cells. In a solid oxide fuel cell (SOFC), the primary structural components include the anode, electrolyte, and cathode. The cathode allows oxygen from the air to enter, while the anode receives fuel (H_2_). The fuel oxidation reaction (FOR) and oxygen reduction reaction (ORR) occur at the anode and cathode, respectively, playing a crucial role in SOFC functionality. The combination of molecular hydrogen and oxygen forms water molecules, representing the fundamental chemical reaction in SOFCs:

2H_2_ + O_2_ = 2H_2_O (1)

**Figure 1:**
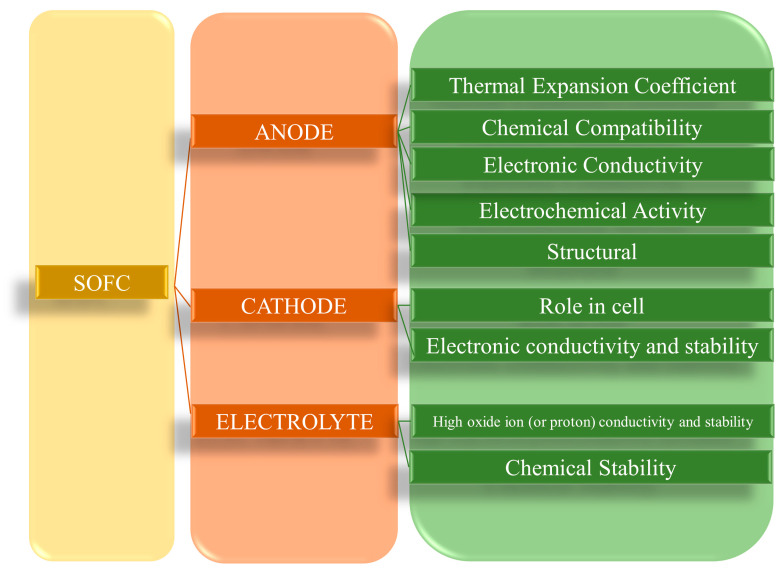
Properties of SOFC components.

Through an external circuit, the electrons created throughout the anodic oxidation reaction are sent to the cathode, where oxygen reduction takes place. The three SOFC components with their various requirements [18] are depicted in Figure 1.

The push for environmentally friendly and sustainable energy generation drives research on replacing hazardous fossil fuels. Among energy converters, solid oxide fuel cells (SOFCs) stand out for their elevated electrical generation efficiency [19]. Compared to other fuel cell types, SOFCs have a number of benefits, such as the capacity to use a variety of fuels, high efficiency, and low susceptibility to fuel contaminants. They also rely on an oxide–ion conducting electrolyte. Because they may employ less expensive catalysts than those based on platinum in PEMFCs and DMFCs, they may also be more affordable [20,21]. The cathode part of SOFCs performs a crucial part in electrochemically removing oxygen. To fulfill this function effectively, the cathode requires specific characteristics: high electronic conductivity (readily exceeding 100 S cm^−1^ in oxidizing conditions), chemical compatibility and matched thermal expansion coefficient (TEC) with electrolyte and interconnecting materials, sufficient porosity for easy oxygen diffusion to the cathode/electrolyte interface, stability throughout fabrication as well as operation under oxidizing conditions, strong catalytic activity for the oxygen reduction reaction (ORR), and cost-effectiveness. Yttria-stabilized zirconia (YSZ) combined with Sr-doped LaMnO_3_ (LSM) is the preferred cathodic material for high-temperature SOFCs, typically those operating between 800 and 1000 °C [22]. Strontium-doped lanthanum manganite (LSM) is considered a highly promising cathode material for high-temperature SOFCs due to its excellent electrochemical performance, thermal and chemical stability, and good compatibility with yttria-stabilized zirconia (YSZ). However, the performance of LSM is limited at lower temperatures due to its low oxygen ion conductivity and high activation energy for oxygen dissociation. The addition of an electrolyte component to LSM in composite cathodes significantly enhances electrochemical performance by extending the three-phase boundary (TPB) areas from the electrolyte/cathode interface into the bulk cathode. Additionally, various mixed ionic–electronic conductors (MIECs) have been employed as cathodes, showing promising results. The electrolyte phase in composite cathodes was commonly reported in the literature to be either doped ceria (DCO) or YSZ, each having pros and cons of its own. While DCO has a significantly higher ionic conductivity but a mismatched thermal expansion coefficient (TEC) with YSZ, YSZ has a relatively low ionic conductivity and matches the electrolyte material well. Typically, three electrodes were used in open-circuit settings to compare LSM/DCO and LSM/YSZ cathodes. Few studies have examined these two cathodes’ realistic fuel cell applications in one study, which would clearly illustrate their value. For example, in an experimental investigation, three types of cathodes were fabricated on anode-supported YSZ thin films to create several single cells: a pure LSM cathode, an LSM/YSZ composite by solid mixing, and an LSM/Sm_0.2_Ce_0.8_O_1.9_ (SDC) composite by ion impregnation. Among these, the highest cell output performance of 1.25 W/cm^2^ at 800 °C was achieved with the LSM/SDC cathode when exposed to stationary air. The highest cell performance of 2.32 W/cm^2^ was obtained with the LSM/YSZ cathode using a 100 mL/min oxygen flow as the oxidant. At reduced temperatures down to 700 °C, the LSM/SDC cathode was the most suitable for zirconia-based electrolyte SOFCs. The variations in cell performance were attributed to the mutual effects of gas diffusion rates and the TPB length of the cathode.

Perovskite-type oxides, as MIEC cathode materials, have been studied extensively for H-SOFCs in recent decades, offering the necessary qualities for an effective cathode electrode across a range of temperatures (500–800 °C) [23]. H-SOFCs have demonstrated significant performance improvements [24,25,26,27], with the power output largely dependent on the oxygen reduction reaction (ORR) kinetics happening near the cathode. Lowering the operating temperature enhances the significance of ORR kinetics [28,29,30], a complex procedure involving the adsorption and dissociation of O_2_ followed by first proton–electron transfer for acidic media and, similarly, the adsorption of O_2_ followed by first and second proton–electron transfer in basic media, which creates water by reducing oxygen molecules at the cathode [31]. The ORR primary response can be shown as follows:

(2) $$\frac{1}{2}O_{2}\;+\;2e^{-}\;=\;O^{2-}$$

O_2_ + 4e^−^ + 4H^+^ → 2H_2_O (3)

The overall rate of the oxygen reduction reaction (ORR) is represented by the total internal resistance, which includes cathode polarization and electrolyte ohmic resistance. This rate is determined by the rate-determining step (RDS), typically the slowest stage in the process. The performance of hydrogen-based solid oxide fuel cells (H-SOFCs) is often limited by the slow kinetics of the oxygen dissociation–absorption process. Understanding these fundamental steps is challenging due to their rapid and complex nature, making direct experimental observation during cell operation difficult. Experimental data can provide insights into the ORR mechanism and the RDS, but first-principles calculations are also valuable for verifying this knowledge from an atomistic perspective [31,32]. In first-principles calculations, a system of atoms is treated as a system of electrons and nuclei. By using interaction theories of nuclei and electrons, various properties of cathode materials, such as molecular structure and energy, can be estimated. Although it is impossible to accurately simulate the full chemical and structural complexity of real oxide surfaces, including chemical inhomogeneities and high-dimensional defects, ab initio calculations based on carefully selected model surfaces can still provide valuable mechanistic information. Combining first-principles calculations with experimental research offers a more comprehensive, though approximate, understanding of the ORR’s complexity. Several recent studies have investigated the pertinent features of various oxide cathode materials using static “density functional theory” (DFT) of electronic states [25,33]. When Adler et al. investigated the ORR process on a porous MIEC cathode, they discovered solid oxygen diffusion along with oxygen surface alteration reactions. They also discovered a relationship between the ORR values and the valuations of D (the oxygen diffusion coefficient) and K (the rate constant of oxygen surface exchange). The Arrhenius equation can be used to describe the value of D:D = A⋅exp [−Q/(kBT)], where the two variables that make up the overall activation energy Q are the activation enthalpy to conduct O_2_ to their closest vacant site and the oxygen vacancy creation free energy. Thus, lower oxygen vacancy generation energies alongside lower migration barrier heights are necessary for high bulk conductivity [34,35]. Breaking the metal–oxygen link, eliminating neutral oxygen atoms, and dispersing the two extra oxygen electrons that remain in the lattice are the sequential steps involved in the production of oxygen vacancies. Important criteria for evaluating the oxygen ion transport properties in materials are the formation energy and oxygen vacancy content. The following formula can be used to calculate the energy (*E_f_*) needed to create an oxygen vacancy:

(4) $$E_{f}=E_{\mathrm{defect}}+\frac{1}{2}E_{O2}\;-\;E_{\mathrm{perfect}}$$

where *E_defect_* is the total sum of energy of the defective bulk, *E_O_*_2_ is the energy of the molecular oxygen, and *E_perfect_* is the total energy of a perfect bulk.

In cathode materials, proton transport usually entails inter- as well as intra-lattice movement as well as hydration (binding to lattice oxygen). One interesting strategy to improve proton conduction in materials is to optimize hydration capacity.

Hydration is the breakdown of H_2_O into OH^−^ and H^+^, where the proton establishes a covalent connection with its adjacent lattice oxygen and the OH group replaces the oxygen vacancy. Therefore, the presence of oxygen vacancies affects oxygen ion migration [36] and promotes proton migration in an oxide. The following formula [37] can be used to determine the hydration energy:

(5) $$\Delta E_{hydration}=E_{2OH}-E_{defect}-E_{H_{2}O}$$

**Figure 2:**
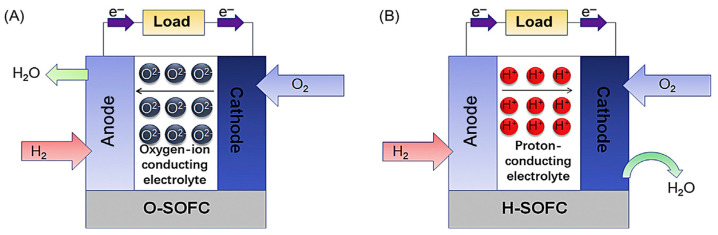
Schematic representation of (**A**) oxygen ion-conducting SOFC–O_2_ and (**B**) proton-conducting SOFC–H^+^ [41].

There are two main phases in the proton migration mechanism: jumping and rotation. The proton jumps from one site attached to an oxygen atom to another oxygen atom that is physically closest to it. The bond between the proton and the oxygen rotates by 90 degrees from its starting position as part of the rotation mechanism [38]. It has been suggested that the proton-leaping phase is the rate-inhibiting phase due to the feeble activation potential for rotational diffusion, as demonstrated by preceding experimental results and quantum molecular dynamics (MD) simulations [39]. A lower potential barrier to proton migration is indicated by lower-level energy. A material is said to have proton conduction capability if the largest energy barrier meant for migration in a given direction is comparable to the threshold of a most general proton-conducting oxide [40]. Proton-conducting (SOFC–H^+^) and oxygen ion-conducting (SOFC–O_2_) electrolytes are the two types found in SOFCs. Figure 2 shows a schematic representation of both SOFC–O_2_ and SOFC–H^+^. Figure 2A illustrates the way the oxygen gas (O^2−^) molecules in SOFC–O_2_ freely pass from the cathode side through the electrolyte and react with the H_2_ gas that is already present at the anode. An oxygen ion reacts with a proton at the anode side to produce steam. Similarly, from Figure 2B, it can be seen that steam is produced and released from the cathode side in SOFC–H^+^ as the hydrogen molecules from the anode pass the anode side and react with the oxygen molecules at the cathode. Voltage is consequently produced by the electrons moving through the external circuit [41].

With the emergence of future energy sources to replace carbon-dependent economies, the drawbacks of the current carbon economy are increasingly apparent [42,43,44,45]. Conventional energy sources like coal, petrol, and diesel emit hazardous CO and CO_2_ emissions globally, underscoring the urgent need for alternatives. Hydrogen, acknowledged as a green energy source, presents promising prospects but also challenges in storage. Storage methods for hydrogen vary, ranging from chemical to physical forms. It can be stored as molecular hydrogen or combined with other elements as hydridic/protonic hydrogen. With a high calorific value of 144 MJkg^−1^, hydrogen offers a cleaner energy alternative, generated through electrolytic water breakdown and fuel cells converting chemical energy into electrical energy. Combining fuel cell and hydrogen storage technologies provides an effective energy transfer solution, with water as a by-product that can be reused for electrocatalysis to produce more hydrogen for future use, making it a potential carbon-free fuel source. Compared to battery-powered vehicles (BPVs), hydrogen-enhanced fuel cell energy storage offers longer storage duration and greater environmental benefits. However, the commercial viability of hydrogen technology remains crucial for developing a carbon-free economy. Despite hydrogen’s low volumetric density and diffusive properties, effective storage solutions are essential. Solid porous materials offer promising options for achieving higher hydrogen storage densities, ensuring safe containment at room pressure and optimal space utilization. The adsorption of hydrogen into solid porous materials primarily occurs through two processes: physisorption and chemisorption. Physisorption, driven by van der Waals forces, is preferred for its thermodynamic stability and reversibility compared to chemisorption, which demands higher temperatures for desorption and exhibits greater irreversibility [46]. The development of efficient and reversible hydrogen storage materials is essential for realizing the full potential of hydrogen as a clean energy source, paving the path for a sustainable and carbon-free energy future [47].

**Figure 3:**
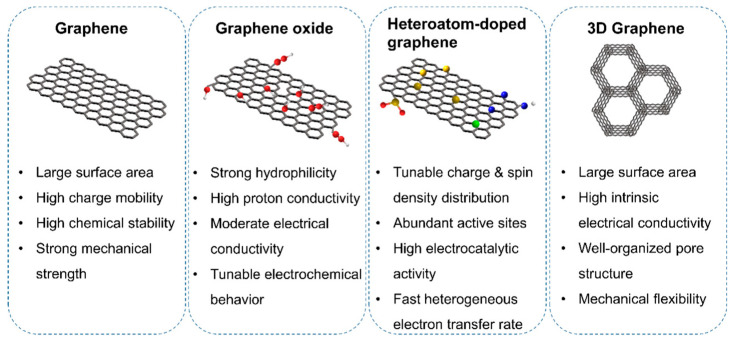
The main properties of graphene-based materials are related to fuel cell applications [46].

Materials based on graphene have been shown to be excellent electrocatalyst substrates, especially in proton-conducting solid oxide fuel cells (H-SOFCs). They are very effective because they can supply a large number of active sites and efficiently transport electrons during oxygen reduction (ORR) and fuel oxidation processes [48,49,50,51,52]. Because of their low cost, excellent resistance to poisoning, and robust electrocatalytic activity, metal-free graphene materials are particularly remarkable. Graphene is an important material in the advancement of this technology since research has shown the enormous influence that defects, doping configurations, alterations to the electronic structure, and functional groups have on the efficiency of H-SOFCs [53,54,55]. Integrating graphene-based materials into proton-conducting electrolytes has been shown to significantly enhance ionic conductivity, thus improving proton transport and reducing fuel crossover. This is crucial for H-SOFCs, where strong proton conductivity and resistance to water, H_2_, and methanol are essential for efficient operation. Graphene-based materials offer the potential to enhance the stability and distribution of fuel/air and improve the current collection of bipolar plates, in addition to their roles in electrolytes and electrodes. Previous studies have extensively investigated the catalytic and electrochemical characteristics of graphene-based materials, with recent evaluations focusing on their application in various components of H-SOFCs, such as ORR, fuel oxidation, membranes, and bipolar plates [56,57,58]. Graphene, a hexagon-shaped sheet of sp^2^-hybridized carbons just one atom thick, serves as the foundation for various derivatives, including functionalized graphene, reduced graphene oxide (rGO), heteroatom-doped graphene, graphene oxide (GO), and three-dimensional (3D) graphene. Renowned for their large specific surface area, exceptional electrical and thermal conductivity, superior mechanical strength, and remarkable chemical stability, these materials possess a plethora of physical and chemical attributes that make them ideal candidates for H-SOFC technology, as displayed in Figure 3 [46].

With their special properties, graphene and its derivatives hold immense potential for advancing H-SOFC technology, contributing to the transition toward sustainable energy solutions. Their ability to improve proton conductivity, enhance catalytic activity, and maintain stability under operating conditions makes them highly suitable for the demanding environment of H-SOFCs. This makes graphene-based materials a promising avenue for future research and development in the field of proton-conducting fuel cells, driving progress toward more efficient and durable fuel cell systems [59].

## 2. Enhanced Catalytic Performance

Investigations of H-SOFCs are now mostly focused on lowering cells’ operational temperatures. Yet, because of a slower ORR activity within the cathode—which is explained by the oxygen molecules’ increased binding affinity at lower temperatures—activation energy becomes greater, reactivity falls, and polarization resistance rises. Numerous investigations have demonstrated that the ORR reaction process’s rate-limiting phases include oxygen exchange as well as bulk/surface diffusion [60]. There have been attempts to investigate and create better ORR cathode catalysts in an attempt to significantly boost the electrochemical properties of H-SOFCs. The catalytic potential of a cathode may be enhanced by a number of parameters, including higher proton, electron, and ion conductivity; more oxygen vacancies; and a longer three-phase reaction interface (TPB), which is the interface connecting the oxygen, electrolyte, and cathode [61]. According to a survey of the literature over the past several decades, the most popular and successful methods for altering the characteristics of two-dimensional materials and enhancing their performance include ionic substitution, the addition of additives, and the creation of certain morphologies [62]. Graphene is highly beneficial in enhancing the catalytic performance of electrodes due to its unique structural and electronic properties to improve catalytic performance. Graphene has an exceptionally high surface area (theoretical value of 2630 m^2^/g) [63]. This provides abundant active sites for catalytic reactions, enhancing the overall catalytic activity. Graphene possesses superior electrical conductivity due to its sp^2^ hybridized carbon atoms arranged in a honeycomb lattice [64]. This facilitates efficient electron transfer during catalytic processes, improving reaction rates. Graphene is chemically stable and resistant to corrosion, which is crucial for maintaining long-term catalytic performance in various environments, including the acidic and basic media commonly found in fuel cells [65]. The mechanical strength and flexibility of graphene make it an ideal support material for catalysts [66]. It can withstand structural stress and maintain integrity under operating conditions, thereby prolonging the life of the catalyst. Graphene’s properties can be easily tuned by functionalization, doping with heteroatoms (such as nitrogen, sulfur, or boron), or creating defects [67]. These modifications can enhance its catalytic activity by altering the electronic structure and creating additional active sites. When used in composite materials with metals or metal oxides, graphene can enhance the dispersion of these catalytic particles, prevent their aggregation, and promote synergistic effects that improve overall catalytic performance [68]. The two-dimensional structure of graphene facilitates the efficient mass transport of reactants and products to and from the active sites [69]. This reduces diffusion limitations and enhances the catalytic efficiency. These properties make graphene-based materials highly attractive for a wide range of catalytic applications, including in H-SOFCs, where efficient HOR and ORR are critical for performance. By improving the activity, stability, and durability of the catalysts, graphene contributes significantly to the development of more efficient and cost-effective energy technologies. This section provides a thorough discussion of methods to increase cathode activity, taking into account both the material’s perceived and intrinsic activity.

### 2.1. Native Catalytic Capability

The efficient performance of an H-SOFC is ensured by the conductivity of oxygen ions, electrons, and protons at an intermediate temperature by improving the proton conductivity of the catalyst perovskite materials. ORR activity occurs in the entire cathode, extending TPB and expanding the reaction sites, where proton conductivity should be approximately 10^−5^ Scm^−1^ to activate a significant area of the cathode surface [70]. In a study of a 3D-SOFC model powered by hydrogen and coal gas for various electrolytes, Kumuk et al. [71] discovered that at intermediate operating temperatures (400–800 °C), protonic ion solid oxide fuel cells (H-SOFCs) outperform oxide ion SOFCs (O-SOFCs), but not at more extreme temperatures (800–1000 °C). The goal of improving the cathode’s proton transport capacity has become a major design goal, leading to the investigation of various novel cathode materials.

Hydrogen molecule adsorption on graphene surfaces follows certain thermodynamics. Since there is a lower degree of disorder when molecules are ordered on graphene layers, the system’s entropy is reduced. Nevertheless, the system’s enthalpy, or total energy, decreases, offsetting the impact of entropy and producing negative free energy (DG). Reduced adsorption stabilities result from the negative TDS term over-dominating when the temperature rises and developing positive free energy. The capacity to adsorb hydrogen is computed as follows:

(6) $$\mathrm{wt}\%\;\mathrm{of}\;\mathrm{Hydrogen}=\frac{M_{H_{2}}}{M_{H_{2}+M_{s}}}$$

where the mass of the sample is *M_S_* and the mass of hydrogen is *M_H2_*, respectively. The procedure of hydrogen adsorption through physisorption in carbon-based compounds including van der Waals contact has been noted by Mohan et al. [72]. Interaction energy is a parameter that measures the physical interactions of solid-state and gaseous materials. It is expressed as follows:

(7) $$E=\frac{\alpha_{H_{2}}\alpha_{\mathrm{Substrate}}}{R^{6}}$$

where R is the interaction distance and α is the polarizability. Since the polarizability of hydrogen gas is determined by the formula above, selecting materials with higher polarizabilities is necessary to improve interaction energy and, consequently, thermodynamics.

According to reports, a standard interaction in the region of 4.5 kJmol^−1^ indicates a weak connection between H_2_ gaseous molecules and carbonaceous solid-state materials [73]. Due to the faster rate at which hydrogen desorbs at elevated temperatures, these conditions are not appropriate for improved storage capacities. Chemisorption is another process where each carbon atom functions as a contact site. Under high pressure, hydrogen molecules split into hydrogen atoms, which close the gap between two nearly adjacent carbon nanotubes (CNTs), facilitating the formation of two C-H bonds. Desorption in this instance also occurs at high temperatures, making it less useful. When the adsorption energy is low, the desorption of hydrogen occurs at lower temperatures, indicating a weaker degree of contact between the solid-state materials and the hydrogen gas molecules. Li et al. described two distinct processes for hydrogen adsorption in solid-state carbon materials [74]. Because of its exceptional physical qualities and distinct two-dimensional structure, graphene has drawn the attention of researchers ever since it was discovered in 2004 [75,76]. Moreover, catalytic support is also thought to be provided by graphene. High catalytic activity on graphene can result from the presence of metal nanoparticles, according to certain research [77,78]. Graphene is not the best support for SACs, though, as the contact between it and the supported metal is often minimal [79,80]. Graphene has been modified with different vacancy defects to improve the interaction between metal atoms and graphene. It is noteworthy that SACs with increased activity can arise from a single Au, Cu, Pt, or Nb atom incorporated in graphene that is faulty [81,82,83,84]. Because of the likely emergence of a cluster distribution of catalyst atoms, it is not desirable for SACs to accurately regulate vacancy generation by atom or ion beam, as this is a challenging experimental problem [85]. It would therefore be ideal to make use of any new tactics that may evenly distribute metal atoms on graphene [86].

#### The Presence of Oxygen Vacancies

Oxygen vacancies, also known as anion defects or “oxygen non-stoichiometry”, occur when the total positive charge of cations decreases due to environmental influences, doping ions (which include metallic and non-metallic ions), or A-site deficiency. Materials with large concentrations of oxygen vacancies, such as BCFN [87], PBC [88], LSCF, and BSCF [89], were studied in the early stages. La_0.6_Sr_0.4_Co_0.2_Fe_0.8_O_3-δ_ was utilized as a cathode in low-temperature O-SOFCs (oxygen solid oxide fuel cells) [90]. It was discovered that physically combining LaSr_3_Fe_3_O_10_ with graphene oxide (GO) improved its ORR kinetics when present in a 0.1 M KOH electrolyte, exhibiting stronger positive half-wave potentials and onsets. The primary function of GO was to act as a good conductive agent to make up for the low conductivity of LaSr_3_Fe_3_O_10_. On the other hand, a two-electron mechanism still dominated the ORR pathway, indicating that the addition of GO did not considerably improve ORR performance. This was probably due to the weak contact that exists between GO and LaSr_3_Fe_3_O_10_. Comparatively, it was discovered that chemically altering a carbon coating layer with a thickness of less than 5 nm on the surface of La_0.6_Sr_1.4_MnO_4_ was a result of a mixed pyrolysis and graphitization process that led to noticeably improved ORR performance, with a diffusion-limiting current density similar to Pt/C and a predominantly four-electron pathway. In addition to enabling electronic conduction that was up to two orders of magnitude faster, the carbon covering provided a greater surface area that improved catalyst–electrolyte interaction. It should be noted that the increased ORR activity of the carbon-coated La_0.6_Sr_1.4_MnO_4_ composite was linked with the intrinsically greater activity of the Mn-containing La_0.6_Sr_1.4_MnO_4_, which was significantly higher than that of the Fe-based LaSr_3_Fe_3_O_10_, in addition to the stronger connection created by chemical synthesis. The strong ORR activity of the double-layered RP manganite LaSr_2_Mn_2_O_7_, which showed a direct four-electron ORR route, provided additional evidence for this claim [91]. By co-doping with more than one metal ion, the concentration of oxygen vacancy, which is essential for proton migration, could be raised, improving the overall characteristics of the material [92].

Graphene oxide (GO) has garnered considerable interest for application in electrochemical devices because of its abundance of oxygen functional groups, huge surface area, strong mechanical qualities, atomic-level thickness, ease of manufacture, and ease of handling. In addition, it has readily changeable features, strong chemical and thermal stability, and environmental compliance [93,94,95]. The highly efficient in-plane proton transmission behavior of GO nanosheets was initially shown by Solangi et al. [96]. GO is made from a sheet-like graphene web that has additional hydroxyl (OH), carboxyl (COOH), carbonyl (C=O), and phenol groups added to its epoxide groups. The presence of hydration and the type of proton carrier that is accessible determine which of the two mechanisms—vehicular or Grotthuss—cause proton conductivity [97,98]. However, because the sp-layered carbons are linked to strong covalent bonds, which reduce their reaction with water and prevent the creation of proton-transmitting hydrophilic channels, GO exhibits a barrier to water uptake. Low proton conductivity is further exacerbated by the non-uniform distribution of the proton-carrying sites within the interlayer GO channels [99,100,101,102]. One of two methods is needed for the GO to be used in a significantly ion-conducting proton exchange membrane fuel cell: either appropriate molecules must be intercalated into the GO or the GO must undergo further chemical functionalization. Both techniques can be used to increase the interlayer’s flexibility and change the water content of GO, which improves the material’s capacity to transport protons [103,104,105].

**Figure 4:**
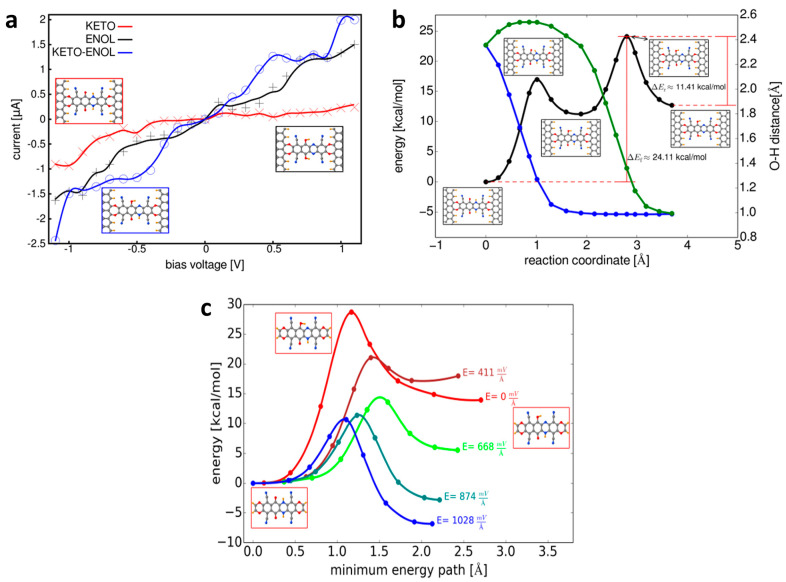
(**a**) Current–voltage characteristics of the single molecule junction with the molecular bridge in the keto (red), enol (black), and keto–enol (blue) forms. The solid lines were obtained by interpolating the transmission functions calculated at different voltages indicated by the symbols +, ×, and O for enol, keto, and keto–enol, respectively. The insets show the different molecular structures of the bridge in the junction (hydrogen atoms are highlighted in orange for clarity). (**b**) Reaction path for the intramolecular proton transfer process in the junction. The black line shows the energy profile along the reaction coordinate and the green and blue lines show the distance of the two relevant protons to the corresponding oxygens. The barrier heights were calculated using the climbing image method. The insets show the geometries as the respective points on the reaction path. (**c**) Reaction path between the keto and keto–enol forms at different values of the external electric field, calculated using a model including only the molecular bridge. The insets show the equilibrium geometries of the keto and keto–enol tautomers and the transition state [108].

By using graphene, an allotrope of carbon, it is possible to get over the experimental restrictions that come with using metal electrodes. Enhancing the experimental monitoring of molecular bridge-bonding has a major effect on the transit of appropriate ties [106,107]. However, the narrow optical absorption cross-section, high thermal conductivity, and ultra-flat two-dimensional shape of graphene make it easier for light to reach the molecular junction, making it possible to pinpoint and manipulate the mechanics of charge transport. In this study, we explored a bonding motif among the various potential designs, where the molecular bridges are covalently bonded to the (metallic-like) zigzag-ended graphene edge. Weckbecker et al. indicated that the conductance mode of a graphene-based molecular junction is controlled by an intramolecular proton transfer process. An applied electrostatic field can influence both the relative stability within the tautomers and the energetic threshold for their interconversion, which makes this process a good contender for a molecular switch. Figure 4 illustrates the sequential mechanism of the intramolecular proton transfer process with an intermediate state of the keto–enol type [108]. Large energy barriers divide the several minima along the reaction path in the absence of an external electrostatic field, preventing the interconversion between the keto and enol states. The current–voltage properties of enol, keto, and keto–enol tautomers are shown in Figure 4a. The findings demonstrate that the three transistors can be employed as the switch’s “on” and “off” states since they realize distinct conductance states across a wide voltage range. In particular, the enol and keto–enol isomers exhibit substantially higher currents in comparison to the keto isomer, indicating that the switch might be in two different “on” states. The bias voltage, which can reach values of up to 28 at 05 V for the keto–enol/keto pair, determines the “on”/“off” ratio and the process’s reaction route as determined by the nudged elastic band (NEB) approach [109,110]. Figure 4b shows the reaction’s minimal energy path as a result. It demonstrates that a stepwise mechanism involving an intermediate state with a keto–enol nature underlies the intramolecular proton transfer process [111]. Large energy barriers divide the several minima along the reaction path in the absence of an external electrostatic field, preventing the interconversion among the keto and enol states. We examined the impact of an external electrostatic field on the keto–enol tautomerization reaction at the molecular level in order to evaluate the viability of efficiently manipulating the conductance state of the junction in a reversible manner. The findings demonstrate the controlled stabilization of several tautomers by an external electrostatic field applied along the molecule’s longitudinal axis. At a threshold field of 800 mV, an inversion of the relative stability of these tautomers was noticed. In addition to this impact, the energy barrier for the conversion of keto to keto–enol was simultaneously modified by the external field. Specifically, the barrier of this chemical phase was lowered to 100 kcal/mol at a field of 1000 mV [112].

According to theoretical research cited in a publication by Shi et al., hydroxyl-terminated graphene provides a significantly lower energy barrier for protonic conduction, and protons can hop more easily when epoxide groups are attached to water molecules [113]. On the other hand, GO returns to high electron conductivity but normal (or low) proton conductivity upon reduction. They discovered that the conductance, whose rate-limiting phases can be either molecule rotation or proton hopping, is explained by the overall drifting of protons through the component of an external electric field orthogonal to the graphene planes. On graphene, the step is the rotation of a water molecule around its connection to an epoxide, at least at ambient temperature, as the dielectric relaxation and conductivity have activation energies that are similar and rise with the concentration of that functional group.

### 2.2. Observable Catalytic Activity

#### 2.2.1. Nanoarchitecture

Enzyme-based nano-fuel cell (EFC) technology has inherent advantages stemming from a high surface-to-volume ratio and improved reactivity [114,115]. However, the practical implementation of such cathodes encounters challenges in maintaining structural integrity with appropriate mechanical strength and stability, often necessitating operating temperatures exceeding 500 °C. At such elevated temperatures, preserving the integrity of the entire cathode nanostructure becomes increasingly impractical, posing a significant hurdle in the advancement of EFCs. A promising strategy for obtaining favorable microstructures and high performance in nanostructured cathodes is the wet impregnation process. Still, questions remain about these materials’ ability to withstand extended exposure to high temperatures. As a result of nanoparticles accumulating at high temperatures over time, catalytic activity gradually decreases. These difficulties highlight the need for different approaches to fully utilize nanostructured cathodes in EFCs. Carbon nanoparticles show promise as a remedy for these problems. These zero-dimensional carbon nanomaterials are excellent candidates for improving EFC performance because of their outstanding surface-to-volume ratio, extraordinary conductivity, simplicity of functionalization, and biocompatibility. Furthermore, because of their innate redox properties, they can function as effective redox intermediates, promoting electron transfer between the surface of electrodes and the redox-active sites of biocatalysts. These characteristics have led to the growing use of carbon nanoparticles and their composites in the development of electrodes for the improvement of EFCs. Researchers want to go beyond the drawbacks of nanostructured cathodes by incorporating carbon nanoparticles into electrode designs. These drawbacks are mostly related to mechanical stability and catalytic activity at elevated temperatures. The application of carbon nanoparticles offers a path toward improved performance, durability, and efficiency and signifies a paradigm leap in EFC technology. Researchers work to open new avenues for the design and optimization of EFCs by utilizing the special qualities of carbon nanoparticles. This will help to facilitate the broad use of EFCs in a variety of applications, such as sustainable energy systems and portable electronics. Carbon nanoparticles combined with nanostructured cathodes offer a promising path forward for electrochemical fuel cell technology. Even though maintaining structural integrity and long-term durability still presents hurdles, continuous research efforts are propelling innovation in this field, pointing to a future in which EFCs will be crucial in accelerating the shift to a sustainable energy future.

**Figure 5:**
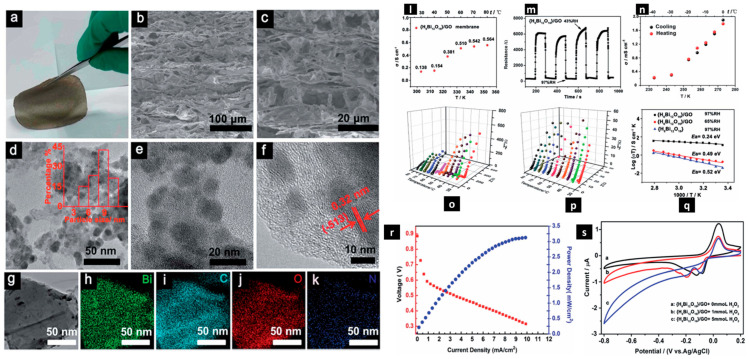
(**a**) The {H_6_Bi_12_O_16_}/GO membrane was obtained via vacuum-assisted filtration. (**b**,**c**) Scanning electron microscope (SEM) cross-sectional images of {H_6_Bi_12_O_16_}/GO. (**d**–**f**) TEM images of {H_6_Bi_12_O_16_}/GO. (**g**–**k**) Corresponding elemental mapping images of Bi, C, O, and N of {H_6_Bi_12_O_16_}/GO. (**l**) Proton conductivity (s) of the {H_6_Bi_12_O_16_}/GO membrane at various temperatures in aqueous solution. (**m**) Typical response curves of the {H_6_Bi_12_O_16_}/GO film upon exposure to 43% RH and 97% RH at 1000 Hz. (**n**) Negligible changes of proton conductivity of humidified {H_6_Bi_12_O_16_}/GO during heating–cooling cycles in sub-zero conditions. (**o**) Nyquist plots for {H_6_Bi_12_O_16_} at 97% RH and various temperatures. (**p**) Nyquist plots for {H_6_Bi_12_O_16_}/GO at 97% RH and various temperatures. (**q**) Arrhenius-type plot of the conductivity of {H_6_Bi_12_O_16_}/GO and {H_6_Bi_12_O_16_} in various temperature and humidity conditions. (**r**) Polarization and power density curves of DMFCs using {H_6_Bi_12_O_16_}/GO/PVA membranes. (**s**) Electrocatalytic reduction of H_2_O_2_ with {H_6_Bi_12_O_16_}/GO in a 0.1 MKH_2_PO_4_–Na_2_HPO_4_ aqueous solution (pH ¼ 7.4) at a scan rate of 50 mV s^−1^ [116].

Liu et al. [116] utilized hydrophilic bismuth oxide {H_6_Bi_12_O_16_} cationic nanocrystals, which are inspired by polyoxometalates, to interact in situ with hydrophilic graphene oxide (GO) to generate a {H_6_Bi_12_O_16_}/GO nanocomposite that is well known for its effectiveness as a proton-conducting material. The composite H_6_Bi_12_O_16_/GO obtained by vacuum-assisted filtration is shown in the accompanying Figure 5, along with images from a scanning electron microscope (SEM) showing its cross-section, images from the transmission electron microscope (TEM), and associated elemental mapping images showing the distribution of bismuth (Bi), carbon (C), oxygen (O), and nitrogen (N). The amazing capacity of this {H_6_Bi_12_O_16_}/GO nanocomposite to overcome the trade-off between proton conductivity and methanol permeability was demonstrated. It exhibited rapid humidity response and recovery qualities, as well as catalytic hydrogen peroxide breakdown and decreased methanol permeability, without sacrificing its high proton conductivity. The {H_6_Bi_12_O_16_}/GO composite membrane had better proton conductivity in an aqueous solution than Nafion, with an in-plane proton conductivity value of 5.64 × 10^−3^ S m^−1^ at 80 °C. Moreover, it continued to function exceptionally well at below-freezing temperatures, recording 2.17 × 10^−2^ S m^−1^ at 40 °C. Interestingly, at 97% relative humidity (RH), {H_6_Bi_12_O_16_}/GO functioned by the Grotthuss mechanism, highlighting its efficiency in proton transport in humid environments.

#### 2.2.2. Three-Dimensional Structure Arrangement

The fact that the attractive forces among graphene sheets cause the graphene sheets to restack, which reduces the number of active sites available, is one of the primary disadvantages of using graphene in fuel cells. Three-dimensionally structured graphene has a customized hierarchical porosity and curved non-planar shape, which can effectively address this problem [117]. Rapid electron movement is made possible by the continuous conductive network, and ion transport is facilitated by the 3D graphene’s interconnected hierarchical porosity [118]. Hence, when used as a conducting support for electrocatalysts, 3D graphene exhibits a higher ORR activity or a quicker HET rate than 3D porous carbon. Furthermore, it is possible to achieve a greater mass loading with a more effective charge transport channel mechanism across the whole framework of 3D graphene electrodes. Graphene spheres, graphene fibers, graphene films that are vertical, graphene networks, graphene cages, and other designs are examples of 3D graphene that result in a variety of electrical, chemical, and mechanical characteristics [119]. For instance, the 3D porous graphene produced using GO laser beam reduction has a broader specific surface area (1520 m^2^ g^−1^) and strong conductivity (1738 S m^−1^) [120]. The decreased gigantic graphene fibers connected by Ca^2+^ demonstrated a remarkable tensile strength of 501.5 MPa and an electrical conductivity of 4.1 × 10^4^ S m^−1^ [121]. The surface layer of the graphene walls of the 3D cabbage-coral-like graphene features a large number of micropores with a standard diameter of 1.6 nm, which is advantageous for electrolyte ion transport [122].

However, while going through the high-temperature sintering procedure, nanoparticles inevitably tend to grain, which causes them to lose their unique morphologies. However, the electrochemical performance of cells can be greatly enhanced by integrating particular morphologies into currently available mature cathode materials. The core–shell [123], nanofiber [124], urchin-like [125], and 3D network structures [126] are some examples of these unique morphologies. Among the prominent morphologies utilized in electrode microstructure optimization is the “core–shell” architecture. This structure keeps the inside nanometal particles from sintering and aids grain refining. In order to optimize oxygen surface exchange kinetics, cathode material possessing a porous 3D microstructure serves to reduce concentration polarization and enhance the functional electrode surface area by adding nano-catalysts over the electrode interface [127]. A core–shell structure was pre-formed as a result of the creation of electrode particles. When enough shell nanoparticles formed an ongoing charge transport pathway, the electrode’s electrical and ionic conductivity rose. In an effort to improve performance, recent research has focused a great deal of emphasis on the cathode’s core–shell structure.

**Figure 6:**
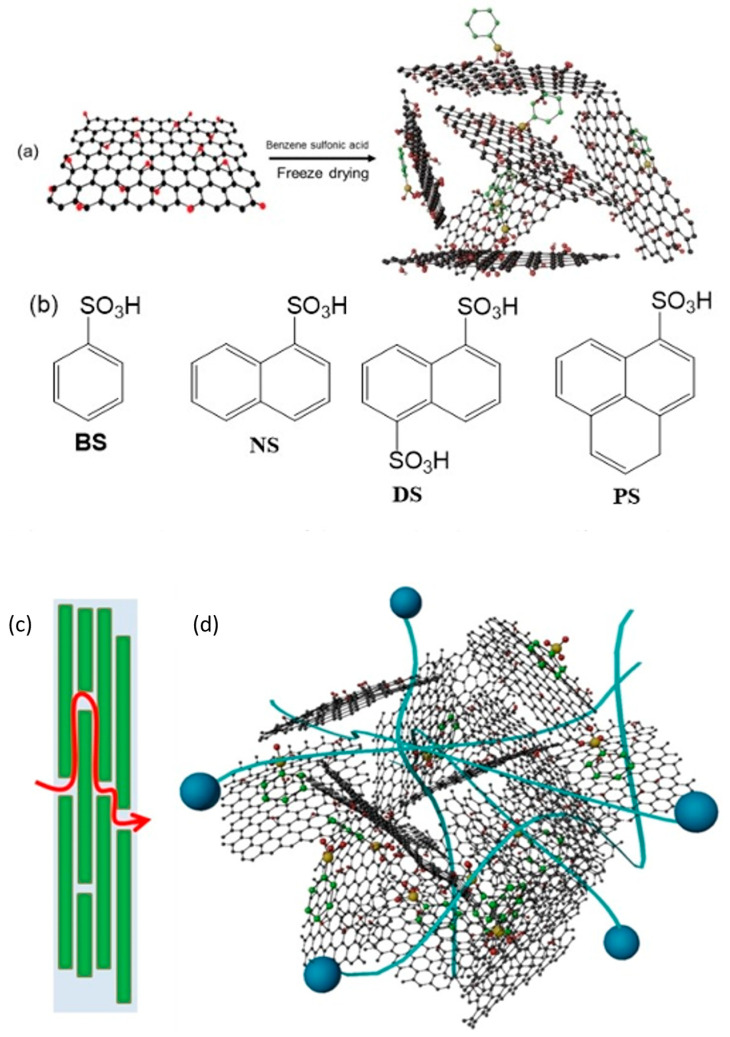
(**a**) Schematic view of the intercalated aromatic sulfonic acid in the interlayer of 3DGO. (**b**) Chemical structure of the aromatic sulfonic acid molecules. (**c**) Mechanism of proton conductivity in 2DGO. (**d**) Mechanism of proton conductivity in 3DGO aromatic sulfonic acid [129].

**Figure 7:**
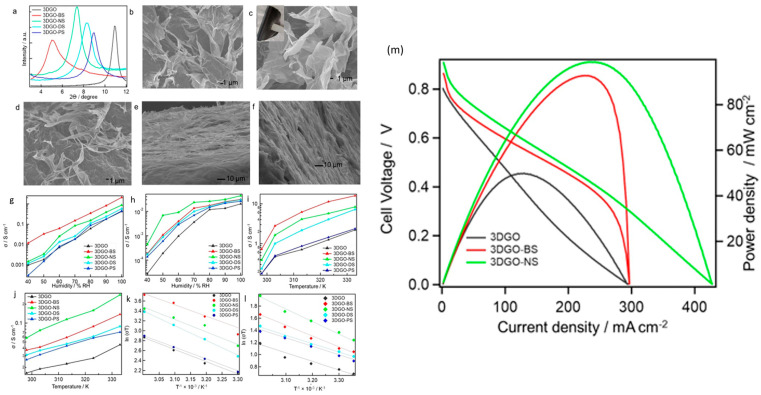
(**a**) Powder XRD patterns of 3DGO, 3DGO-BS, 3DGO-NS, 3DGO-DS, and 3DGO-PS and SEM images of (**b**) unpressed 3DGO, (**c**) unpressed 3DGO-NS (the image is of a section of curved membrane detached by tweezers), (**d**) unpressed 3DGO-BS, (**e**) a cross-section of pressed 3DGO, and (**f**) a cross-section of pressed 3DGO-NS showing the proton conductivities of 3DGO aromatic sulfonic acid derivatives. (**g**) In-plane proton conductivity under different humidified conditions at 25 ° C. (**h**) Out-of-plane proton conductivity with varying humidity at 25 ° C. (**i**) In-plane proton conductivity at different temperatures under 90% RH. (**j**) Out-of-plane proton conductivity at different temperatures under 90% RH. Presented as Arrhenius plots of ln(σT) vs. T^−1^ for (**k**) in-plane direction. (**l**) Out-of-plane direction measured at 90% RH. (**m**) Variation of cell voltage and power density with current density for 3DGO, 3DGO-BS, and 3DGO-NS membranes for a fuel cell operating under 100% RH at 30 °C [129].

Since 3D graphene oxide (3DGO) has a very porous structure and offers several pathways for protons to propagate in diverse directions, resulting in a greatly reduced mean proton conduction pathway, its usage has appeared to be particularly intriguing [128]. It can be very difficult to guarantee a long lifespan for a PEMFC while still keeping its high performance, particularly in elevated temperature and low humidity environments. According to a different method, PEMFC effectiveness can be increased by adding a dopant that has both hydrophobic and hydrophilic domains, such as aromatic sulfonic acids, as shown in Figure 6. Consequently, the hybrid’s water content rises when sulfonic acid functionality is present, and the hydrophobic aromatic group greatly enhances the film’s mechanical strength [129]. The proton transmission behavior of composite 3DGO components is described as occurring after their individual incorporation alongside benzene sulfonic acid (BS), naphthalene sulfonic acid (NS), naphthalene disulfonic acid (DS), and pyrene sulfonic acid (PS), according to a study by Rahman et al. Figure 7a displays the PXRD scatters of the 3DGO variations. A peak is seen in the 3DGO pattern at 9.46°, with an intermediate layer separation of 0.93 nm. Their PXRD patterns show how the derivatives’ corresponding interlayer distances changed. The XRD peak of the membrane shifted to lower angles during doping with aromatic sulfonic acids. The dimension as well as the direction of the interjected organic molecules affected the GO interlayer distance. Figure 7b–f displays optical pictures of 3DGO, 3DGO-NS, 3DGO-BS, 3DGO-DS, and 3DGO-PS. SEM pictures show that, the structures of the 3DGO derivatives seem to be pliable, akin to clay. There is no proof that there was any major structural instability after the introduction of the aromatic sulfonic acid molecules because both 3DGO along with the aromatic sulfonated derivatives exhibited crumpling features, which presumably indicates identical textures. Figure 7g displays the in-plane as well as out-of-plane proton conduction for 3DGO along with its variations, which were measured under various experimental setups. The results were generated by estimating the semi-circular contours of the respective Nyquist plots. As predicted, it is clear from the graphs in Figure 7h that at 25 °C, the corresponding proton conductivities rose alongside higher relative humidity (RH). Even at lower humidity levels, 3DGO-NS demonstrated effective proton conduction for the humidity-dependent proton conductivity in the out-of-plane direction. At 40% relative humidity, 3DGO showed a proton conductivity of 2.8 × 10^−3^ Sm^−1^. Proton conductivity was 2.0 Sm^−1^ at 100% relative humidity. The proton conductivity of 3DGO-NS was measured at 40% RH and found to be 4.4 × 10^−2^ Sm^−1^; when RH increased, this value increased as well, with 4.4 Sm^−1^ being the optimal value. In the instance of 3DGO-BS, the proton conductivity in the out-of-plane direction was determined to be 1.8 × 10^−2^ Sm^−1^ at 40% RH and 3.1 Sm^−1^ at 100% RH. For the purpose of assessing each prepared film’s potential use in PEMFCs, single-cell performance was examined. Figure 4 displays the I-V and current–power density graphs for 3DGO, 3DGO-NS, and 3DGO-BS. Using both the 3DGO-NS and 3DGO-BS membranes resulted in a notable boost in cell performance. An ideal current density and power density of 2318.3 Am^−2^ and 987.6 Wm^−2^ were noted for 3DGO-NS. In addition, 3DGO-BS provided a maximum current density of 2268.2 Am^−2^ and a high-power density of 927.5 Wm^−2^. In contrast, the PEMFC performance using a 3DGO membrane produced a current density of 1205.7 Am^−2^ and a power density of approximately 500 Wm^−2^ under the same circumstances. Thus, the power density attained for the 3DGO system was around twice that obtained for the 3DGO-NS and 3DGO-BS systems. It is obvious that the increased proton conduction capabilities of the 3DGO-NS and 3DGO-BS systems accounted for the observed improved performance. Furthermore, 3DGO-NS and 3DGO-BS have open-circuit voltages (OCVs) of approximately 0.9 V or greater, indicating that our produced membranes have considerable resistance to fuel penetration [130,131,132]. Moreover, the thickness of the membrane has a significant impact on how well a fuel cell operates; thinner membranes are linked to better performance. Vacuum-assisted two-dimensional graphene oxide (2DGO) performs less well than three-dimensional graphene oxide (3DGO). This is because the restacking of the graphene oxide nanosheet results in a longer proton conduction channel with a significant decrease in proton conductivity (Figure 6b). Conversely, a multitude of routes including comparatively shorter ion channels enhance this membrane’s ability to conduct ions (Figure 6c). The large surface area, high porosity, and interconnected pore structure of the 3DGO membrane not only enhance mass transport to the membrane but also offer more pivotal locations for proton-to-proton exchange [133].

## 3. Exceptional Long-Term Durability

Because of their intricate structure, SOFC systems are susceptible to performance degradation in several subsystems and components. Subtle problems like increasing impedance and air leakage might develop over time and cause more concerns [134]. The deterioration of performance is mostly caused by the loss of thermal and chemical stability. A number of degradation processes cause H-SOFCs to perform worse as the running duration grows. It is difficult to achieve stability over time in SOFC systems; this is because strong components and layouts that can endure extreme operating conditions are needed, as well as an understanding of degradation mechanisms. The goal of ongoing research is to overcome these obstacles and raise the long-term reliability of SOFC systems [135].

### 3.1. Chemical Durability

Chemical modification makes it possible to tie various groups to the GO surface, changing the material’s capacity to absorb water. GO’s mechanical strength is significantly reduced as a result of its chemical functionalization [136]. According to research by Matsumoto et al., the addition of sulfate ions to a graphene oxide (GO) membrane produces a proton conductivity of roughly 10^4^ mSm^−1^ [137], which is roughly double that of an undoped GO membrane. Furthermore, the intercalation of other acids, such as phosphoric or formic acid, also causes a significant increase in ion conduction within the resulting GO membranes, as described by Hatakeyama et al. [138]. Researchers also improved the ion-conducting behavior of GO by intercalating aromatic sulfonic acids into it; nonbonding π–π stacking interactions stabilized the resultant hybrids [139]. The achieved power density when employing graphene oxide (GO) as a solid electrolyte is still low when compared to other materials used, despite years of research focused on optimizing GO proton conductivity. This illustrates how closely cell performance is correlated with the membrane’s capacity to carry protons out-of-plane. In other words, proton transmission through layers of GO nanosheets is impeded by their stacking configuration [140].

### 3.2. Stability under Thermal Conditions

#### 3.2.1. Chemical Modification through Doping

There are countless opportunities for alteration and functionalization due to graphene’s flexible carbon structure. One useful method for adjusting the electrochemical and catalytic characteristics of graphene is to dope heteroatoms (for example, nitrogen, boron, sulfur, halogens, or transitional metals) into the basal plane and reactive edges simultaneously. Its electrical characteristics can be changed by further structural flaws and the impact of dopants that donate or withhold electrons [141]. Additionally, these dopants alter the conductivities of materials made from graphene by altering the density of state (DOS) close to the Fermi energy level. Additionally, heteroatom doping results in a shift in the atomic charge distribution and spin density distribution, which increases graphene’s active spots and greatly improves catalytic activity [142]. The most common dopant added to graphene is nitrogen. Nitrogen dopants come in different forms, such as nitrogen oxide, quaternary-N, pyridinic N, pyrrolic N, and amino. The doping N atom united into a hexagonal ring is known as the quaternary-N or graphitic N. By contributing one or two p electrons to the π system, pyridinic and pyrrolic N create sp^2^ and sp^3^ hybridized bonds, respectively. Since nitrogen has a greater electronegativity value (3.04) than carbon atoms (2.55), the surrounding carbon atoms have a positive charge density. Substitutional nitrogen lowers the DOS around the Fermi energy level, leading to bandgap opening. Increased HET rates are also seen for Fe(CN)_6_^3−/4−^ probes in N-doped graphene [143]. Additionally, N-doped graphene resists chemical oxidation more steadily. However, because of the generated defects and hindering effects of dopants, N-doped graphene has poorer carrier mobilities and conductivity than pristine graphene.

#### 3.2.2. Integrated Cathode Materials

**Figure 8:**
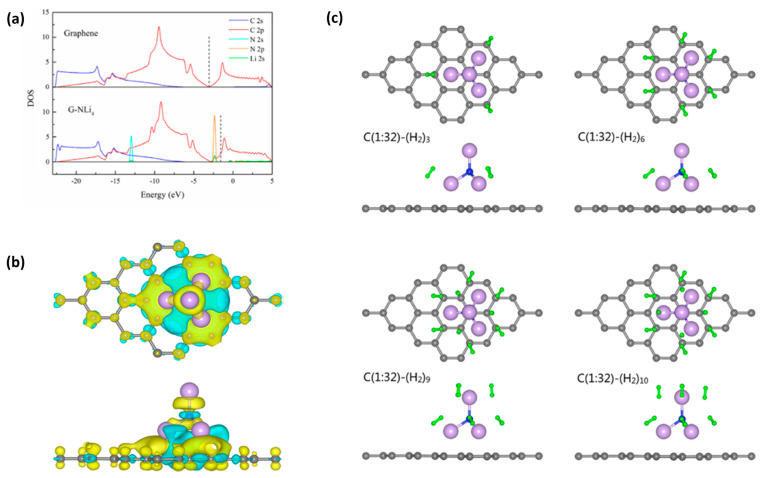
(**a**) Density of states in pristine and NLi_4_-decorated graphene; the Fermi levels are marked with dashed lines. (**b**) Charge density difference of NLi_4_-decorated graphene. Blue and yellow areas indicate electron depletion and accumulation, respectively. (For interpretation of the references to color in this figure legend, the reader is referred to the web version of this article.) (**c**) Top and side views of the optimized configurations of a NLi_4_ unit anchored on graphene. NLi_4_ cluster is situated above the C atom and the three bottom Li atoms are situated above the carbon rings [144].

**Figure 9:**
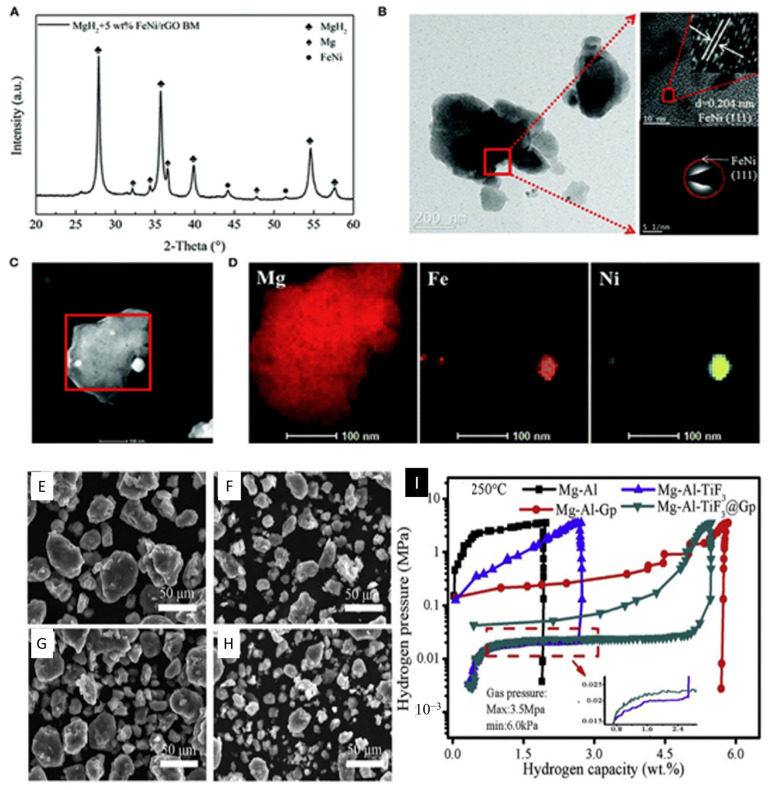
Structural characterization of ball-milled MgH_2_-5 wt.% FeeNi/rGO. (**A**) XRD pattern. (**B**) TEM photograph with the HRTEM image and SAED pattern. (**C**,**D**) Corresponding EDS spectra with elemental mapping of Mg, Fe, and Ni. SEM images of the MgeAl alloy and MgeAl-M (M ¼ GS, TiF_3_, TiF_3_@GS): (**E**) MgeAl, (**F**) MgeAl-GS, (**G**) MgeAleTiF_3_, (**H**) MgeAl-TiF_3_@GS, and (**I**) PCT curves at 250 °C [145].

**Figure 10:**
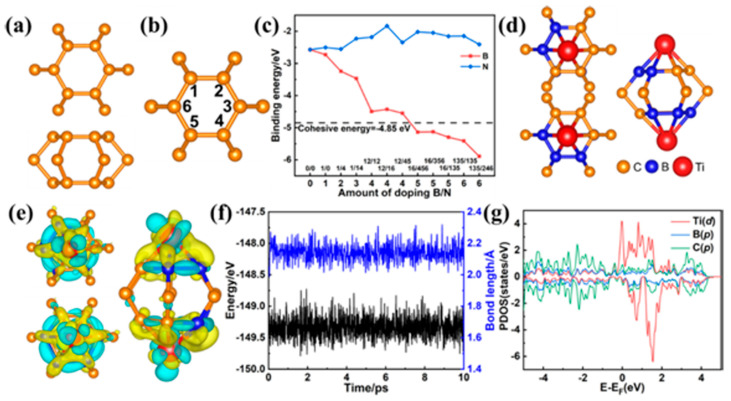
(**a**) Top and side view of twin graphene. (**b**) Names of doping positions. (**c**) Binding energy of different amounts of B/N doping. (**d**) Top, bottom, and side view of the adsorbent 16/456. (**e**) Charge density difference of 16/456 (contour lines in plots are drawn at 0.01 e/Å^3^ intervals). (**f**) Changes in energy and bond length in ab initio molecular dynamics simulation (300 K, 10 ps); the bond length is the average bond length of the Ti-B bond and the Ti-C bond. (**g**) Projected density of state of 16/456 [146].

**Figure 11:**
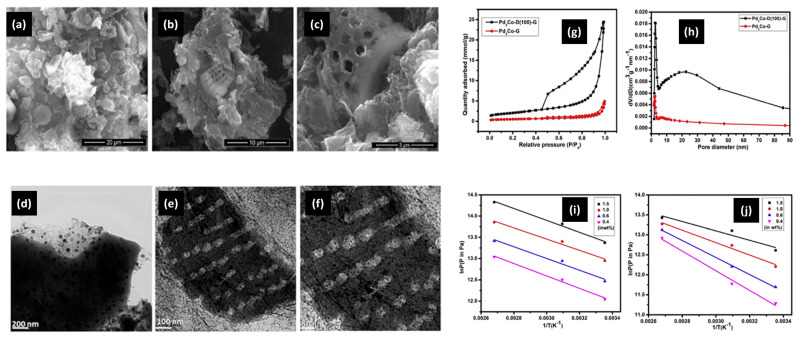
(**a**–**c**) Scanning electron micrographs of Pd_3_Co-D(100)-G. (**d**–**f**) Transmission electron micrographs of Pd_3_Co-D (100)-G. (**g**) Nitrogen adsorption isotherms at 77 K of Pd_3_Co-G and Pd_3_Co-D(100)-G. (**h**) BJH pore size distribution of Pd_3_Co G and Pd_3_Co-D(100)-G. Adsorption isosters of (**i**) Pd_3_Co-D(50)-G and (**j**) Pd_3_Co-D(100)-G for different adsorbed amounts of hydrogen [147].

**Figure 12:**
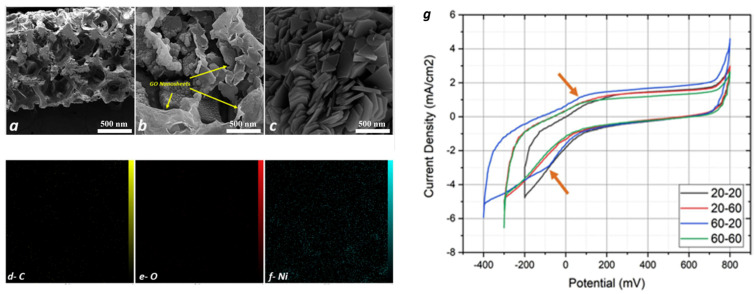
FE-SEM images of (**a**) cross-section, (**b**) morphology of applied GO layer, and (**c**) flower-shape morphology of applied GO layer. MAP distribution of (**d**) carbon, (**e**) oxygen, and (**f**) nickel elements. (**g**) CV curves of GO-coated samples at 100 mV/s with a 6 M KOH solution at room temperature [148].

In order to determine hydrogen storage properties, Qi et al. first constructed the super alkali NiLi_4_ over graphene surfaces and carried out first-principles calculations [144]. The molecular architecture based on super alkali atomic clusters prevented metallic atoms from clumping together and increased the number of sites available for hydrogen adsorption (Figure 8). NiL_4_ atomic clusters have the potential to adsorb up to ten hydrogen molecules per cluster, theoretically. Conversely, NiL_4_-enhanced graphene exhibits a mean adsorption energy value of 0.21 eV per hydrogen molecule and a hydrogen capacity of 10.75 weight percent. Theoretically, this kind of composite substance demonstrated extremely recoverable hydrogen storage capabilities. With an emphasis on easy availability, the storage capacity of hydrogen, the high reversibility of hydrogen with adsorption and desorption capacities, and low cost, Feng et al. reviewed magnesium-based alloys for hydrogen storage (Figure 9) [145]. However, they also noted some disadvantages, including feeble kinetics with greater thermodynamic stability. Because of their exceptional structural, electrical conduction, and magnetic properties, transition metals are the finest choice for catalysts in the desorption or dehydrogenation of alloys. Because of its unique sp^2^-bonded hybrid structure, great chemical stability, and maximum surface area of 2.6 × 10^5^ m^2^kg^−1^, 2D metal graphene was used as a substrate for transition metal catalysts. In this work, the intrinsic process of graphene serving as a catalyst for magnesium-based hydrogen-storing alloys was rigorously examined (Figure 10). It was deduced that in Mg-based alloys, graphene serves as a catalyst, co-catalyst, and preventive measure against the formation of accumulated grains in big aggregates. Dong et al. theoretically developed an ideal hydrogen conservation material based on the boron-doped twin graphene that is patterned with a transition metal through the use of first-principles calculations [146]. Theoretically, it was determined that the bonding strength among the transition metal with the underlying assistance strengthens with an increase in the number of boron-dopant atoms. However, as this work has shown, the hydrogen adsorption capacity gradually decreases, for instance, in Ti-patterned, B-doped, twin-graphene, carbon-based storage compounds (Figure 11). Using graphene as the material G and diatom frustules as the material D, Samantaray et al. created a nanomaterial composite called Pd_3_Co-D(100)-G that had a pore volume of 0.84 cm^3^g^−1^ and a surface area of 163.25 × 10^3^ m^2^kg^−1^ [147]. Due to the unique characteristics of diatom frustules (D), which have a good amount of pore volume and are chemically nonreactive, as well as the adsorption characteristics of the high amounts of hydrogen present in transition metals as well as their respective alloys, the composite nanomaterial was able to store approximately 4.83 weight percent of hydrogen at a temperature of T = 25 °C and had a balancing pressure of hydrogen of 20 bar. The study concentrated on the important finding that increased pore volume and surface area can greatly boost room-temperature hydrogen storage capabilities at comparatively feeble hydrogen pressures. By putting graphene oxide on Ni foam at varying supplied potentials of 20 V and then 60 V for varying times of 20 min and 60 min, Bonab et al. deposited material [148] electrophoretically. Under ideal circumstances, the graphene oxide layers’ morphological characteristics were shown by field emission scanning electron microscopy (FESEM) (Figure 12) or rosette flowers. After applying a potential of 60 V for 20 min, the electrophoretically deposited layer’s optimal hydrogen storage capacity of 50.9 Ah kg^−1^ was attained.

Pd-based nanoflowers (NFs) upon phosphomolybdic acid-modified graphene were reported by Fan et al. [149] for FAOR showing an 8-fold greater maximum current density (1.02 A mg^−1^) compared to that of Pd nanoparticles/graphene counterparts (0.13 A mg^−1^). The strong CO-oxidizing capacity of phosphomolybdic acid (HPMo); the huge interfacial area with stronger binding among Pd, phosphomolybdic acid, and graphene; and the abundance of defects in the nanoflower shape were all credited with the higher activity. The durability of palladium (Pd) is also significantly affected by its shape. Among the various shapes, the nanoflower configuration demonstrates much higher durability compared to the nanosphere. This enhanced durability is further increased when HPMo is introduced. The combination of Pd nanoflowers with HPMo (Pd NF/HPMoG) results in a slight morphological change, which provides additional evidence of its superior durability. Since durability and degradation rate are inversely related, the high durability of Pd NF/HPMoG corresponds to a lower degradation rate. This implies that Pd in the nanoflower form, especially when combined with HPMo, degrades more slowly than in other forms, thus maintaining fuel cell functionality for a longer period. The utilization of F-doped graphene to assist rhodium nanoparticles is a further intriguing layout strategy for FAOR. The stability of 136 Rh/F-doped graphene was far greater than that of a Pt/C catalyst, which only retained 40% of its activity over 100 cycles. Feng et al. [150] mounted core–shell Au@AuPd on rGO and found that, thanks to the complementary actions of core–shell nanostructures with rGO facilitation, it performed better at oxidizing glycol than the Pd/C catalyst. Hu and colleagues created a metal-free 3D graphene anode meant for the carbohydrazide oxidation reaction. Because of its greater surface area and stronger electrical conductivity, this anode demonstrated a two-fold increase in activity when compared to 2D graphene [151]. An optimal power density of 249 W m^−2^ was produced by an anion exchange membrane fuel cell (AEMFC) with 3D graphene as the anode and N-doped carbon nanotubes as the cathode. The performance metrics for various graphene-based materials are compared in Table 2.

## 4. Conclusions and Perspectives

For fuel cell applications, single-layer graphene and graphene oxide (GO) often lack the required proton conductivity. However, the incorporation of graphene-based fillers in polymer membranes can provide a trade-off for proton conductivities and barrier qualities. Careful optimization of the loading amount is necessary, as excessive amounts of GO and functionalized graphene may hinder the proton-conductive channels within the polymer membrane. Further research is needed to evaluate the different functional groups, topologies, and compatibility of graphene-based fillers with polymers and how these factors influence fuel cell performance, impermeability, and proton conductivity. Graphene holds significant promise for fuel cell applications due to its atomic-level availability, high degree of electrochemical activity, and low resistance to ionic transfer diffusion to electrode surfaces. The activation of graphene surfaces through chemical functionalization and doping is crucial to enhance its performance. This process is necessary because physical adsorption through π-π contact is the primary mechanism for associating other molecules with graphene surfaces, especially those with high crystallinity levels. The altered electrical characteristics of graphene sheets are also essential for the development of electrochemical devices. Moreover, graphene offers advantages such as excellent mechanical properties, low weight, and a large surface area that can be optimized for adsorption sites. Graphene’s chemically preferred adsorption properties make it suitable for the long-term transportation or storage of adsorbates, such as hydrogen, which is preferred over conventional bulky and hazardous storage methods like metal hydrides. In transitioning towards a hydrogen-based economy, an enhanced solid-state storage system incorporating nanomaterials along with nanocomposites, including graphene, is appealing. While metal hydrides offer chemical storage solutions, their high cost and unfavorable reversibility properties limit their practical use. Nanomaterials and nanocomposites, on the other hand, provide high storage capacities due to their unique composition and large surface area. However, challenges such as data reproducibility, instrumentation errors, hydrogen spillage, and environmental impacts necessitate thorough investigations and life cycle assessments of hydrogen storage fuel cell devices. Considerations like toxicity, greenhouse gas emissions, scalability, solvent recyclability, and water purification are crucial when employing graphene for hydrogen storage on a large scale.

## Figures and Tables

**Table 1:** Detailed comparison of fuel cell types, performance, operating conditions, electrolytes, and longevity.

Fuel Cell Type	Power Range	Temperature Range (°C)	Typical Fuel	Electrolyte Type	Efficiency	Life Span (h)	Ref.
SOFC	≤1 MW (up to 250 kW)	500–1000	Hydrogen, Methanol, Hydrocarbons	Porous Ceramic Material	50–60%	20,000–80,000	[10]
PEMFC	≤1 MW (up to 200 kW)	110–180	Hydrogen	Water-based Polymer Membrane	45–55%	60,000–80,000	[10]
PAFC	≤11 MW (100–400 kW)	150–220	Hydrogen, LNG, Methanol	Phosphoric Acid	30–42%	40,000–60,000	[11]
AFC	≤500 kW (up to 200 kW)	60–200	Hydrogen	Potassium Hydroxide	40–50%	5000–8000	[12]
MCFC	≤1 MW (up to 250 kW)	650–800	Hydrogen, Methanol, Hydrocarbons	Molten Carbonate Salt	43–55%	15,000–30,000	[11]

**Table 2:** Various graphene-based materials and their efficiency in hydrogen storage.

Graphene-Based Cathode Material	Performance	Ref.
NiLi_4_ graphene	storage capacity of hydrogen, high reversibility of hydrogen, 10.75 wt% hydrogen storage	[144]
Graphene-catalyzed Mg-based hydrogen storage alloys	high energy density, high hydrogen storage capacity, fast hydrogen uptake and discharge kinetics, low thermodynamic stability	[145]
Boron-doped twin graphene	improved hydrogen storage capacity (gravimetric density of 4.95 wt%)	[146]
Au@AuPd-rGO	enhanced electrocatalytic activity and durability	[150]
LCZ oxide graphene	power density of 2675 W m^−2^	[152]
GO La_0.3_Sr_0.7_Fe_0.4_Ti_0.6_O_3-δ_	power density of 362 mWcm^−2^, specific resistance of 0.02 × 10^−4^ Ωm^2^	[153]
Pd_3_Co-D(100)-G	pore volume of 0.84 × 10^−6^ m^3^g^−1^, surface area of 163.25 m^2^g^−1^	[147]
GO Ni foam	hydrogen storage capacity of 50.9 Ah kg^−1^	[148]

## Data Availability

No new data were created or analyzed in this study. Data sharing is not applicable to this article.
